# Prediction Accuracy of Hyperelastic Material Models for Rubber Bumper under Compressive Load

**DOI:** 10.3390/polym16172534

**Published:** 2024-09-07

**Authors:** Dávid Huri

**Affiliations:** Department of Mechanical Engineering, Faculty of Engineering, University of Debrecen, H-4028 Debrecen, Hungary; huri.david@eng.unideb.hu

**Keywords:** rubber bumper, finite element analysis, material models, hyperelasticity, Mooney-Rivlin, Yeoh, curve fitting, Drucker’s stability

## Abstract

Different hyperelastic material models (Mooney-Rivlin, Yeoh, Gent, Arruda-Boyce and Ogden) are able to estimate Treloar’s test data series containing uniaxial and biaxial tension and pure shear stress-strain characteristics of rubber. If the rubber behaviour is only determined for the specific load of the product, which, in the case of rubber bumpers, is the compression, the time needed for the laboratory test can be significantly decreased. The stress-strain characteristics of the uniaxial compression test of rubber samples were used to fit hyperelastic material models. Laboratory and numerical tests of a rubber bumper with a given compound and complex geometry were used to determine the accuracy of the material models. Designing rubber products requires special consideration of the numerical discretization process due to the nonlinear behaviours (material nonlinearity, large deformation, connections, etc.). Modelling considerations were presented for the finite element analysis of the rubber bumper. The results showed that if only uniaxial compression test data are available for the curve fitting of the material model, the Yeoh model performs the best in predicting the rubber product material response under compressive load and complex strain state.

## 1. Introduction

Rubber-based machine elements, like vibration dampers, bumpers, and seals, are becoming increasingly common due to synthetic rubber compounds’ development and shock-absorbing behaviour. In the automotive sector, they perform a crucial function in stabilizing the engine, transmission or cabin, as a chassis component, or as a jounce bumper that limits the last stage of movement. Even with the advancements in electromobility, many of these components are still required in large quantities during the manufacture of automobiles. However, because of the modified structural designs, products must be redesigned considering the loads resulting from the altered total vehicle weight. The only way to satisfy the resulting market demands in time involves innovative design processes that use optimization and the finite element method to determine the rubber product’s design [1,2,3,4,5,6,7,8,9,10]. Since a large portion of the literature [11,12,13,14,15] works on the numerical analysis of rubber bumpers, the research aimed to determine which considerations have to be taken to accurately model the behaviour of these rubber products under compressive load.

Rubber belongs to the group of elastomers, i.e., a polymer with elastic and viscous properties. When modelling rubber products, it is necessary to deal with nonlinearities due to material, geometric and contact effects. Numerical solvers for nonlinear equation systems can be found in commercial finite element software packages. Selecting a calibrated material model that describes the stress-strain relation is necessary for a structural analysis. The material response can be derived from either the phenomenological modelling of measurable macroscopic quantities like the relationship between stress and strain or the micromechanical modelling of local material behaviours and interactions.

A hyperelastic constitutive model that describes the relationship between stress and strain using relevant continuum mechanics theories and the strain energy density function can predict the nonlinearly elastic and isotropic behaviour of rubber materials [16,17,18]. In phenomenological hyperelastic material models, the material response is approximated by mathematical functions. Mooney-Rivlin [19,20], Ogden [21], Yeoh [22], Arruda-Boyce [23] and Gent [24] are a few examples of these model formulas; the latter two were derived using micromechanical model approximations. The load mode, the raw material, or its availability in the finite element software are the main factors that influence the choice of material model [25]. Since the rubber compound used in the bumper is a trade secret, raw material measurements for various load cases are always required. Using these data, a process called curve fitting can be used to determine the values of the parameters included in the constitutive equations [26,27].

The combination of the uniaxial, biaxial or simple shear test will enhance the characterization accuracy of the hyperelastic behaviour of the rubber. Since only the uniaxial test is standardized, most material testing laboratories only have the equipment needed to perform this test. Treloar’s natural rubber measurement data set [28] is used extensively in the literature [29,30,31,32,33] to evaluate the accuracy of material models. Examining twenty distinct material models was performed in one of the most often cited works. While the Mooney-Rivlin model performed well for a restricted strain of 250%, the Ogden model was among the best at describing the behaviour of the material over the whole deformation range [31]. Forty-four different hyperelastic material models were fitted using a multi-objective optimization process based on a genetic algorithm. The number of material parameters was considered when selecting the best-performing model, in addition to the goodness of fit [32]. Based on an evaluation system that considers the standard deviation of the approximation and the number of material parameters, a ranking of fifteen hyperelastic models was presented. The results show that the Ogden and Yeoh model is one of the best-performing models [33]. In [34], 85 different isotropic strain energy function-based material models were fitted for Treolar’s unfilled and Yeoh’s filled rubber data set. The fitting results showed that the two data sets’ best-performing material models are different. Numerical stress solution method was used to calibrate hyper-visco elastic solids subjected to various loading modes in [35,36,37], and prediction capability was tested by simulation and measurement of the radial force in an aged seal [38]. Few studies have tested the accuracy of the fitted material model using rubber product finite element simulation [39,40,41]. After a thorough analysis of 25 hyperelastic constitutive models, Steinmann and Hossain [29,30] concluded that when just one type of test data is available, the prediction of the natural rubber behaviour under the remaining two load modes deviates irregularly using the different constitutive models. In the lack of material test results, a satisfactory alternative could be the inverse material calibration. Nevertheless, it is a difficult and computationally demanding task, particularly when dealing with numerous material parameters [42,43,44].

Based on the reviewed literature, there is enough knowledge available about the behaviour and relative accuracy of hyperelastic material models fitted to the Treloar measurement data set. These showed that the Mooney-Rivlin, Ogden, Yeoh, Arruda-Boyce, and Gent were the most accurate phenomenological models. However, one of the engineering applicability problems of these results is that, in the case of rubber products under compression, the strain range is smaller than the tested one, and the Treloar data set does not contain uniaxial compression measurement. The rubber bumper material is a blend of natural rubber (NR) and styrene-butadiene rubber (SBR), which differ from the natural rubber used in the Treolar dataset and may have an impact on the prediction accuracy of the different hyperelastic material models. To numerically calculate the operating characteristics of a rubber product under compressive load, it is vital to select an appropriate hyperelastic material model and to be aware of its accuracy. It is often possible to identify the primary load and its extent based on the design requirements. In addition, a measure for the element strain state called biaxiality indication will be used to determine the load mode required for the curve fitting of the hyperelastic model. In the case of the investigated rubber bumper, this is the uniaxial compressive load up to a 30% drop in height. None of the reviewed studies examined the accuracy of predicting the behaviour of a rubber product under compressive load if only the uniaxial compression data set is used for the material model calibration. Consequently, this research aim was to fit various hyperelastic material models (Mooney-Rivlin, Ogden, Yeoh, Arruda-Boyce, Gent) for the stress-strain characteristics derived from the uniaxial compression test. Furthermore, the study sought to evaluate the accuracy of the fitted material model using the rubber bumper finite element simulation for compressive load.

## 2. Hyperelasticity

If we ignore the viscoelastic behaviour of the rubber, then its stress state depends on the current state and any stress measure depends on the deformation gradient F. The strain energy density function is used by a hyperelastic constitutive model to describe the stress-strain relation [16,18]. If the strain energy is only a function of the initial and instantaneous state, i.e., it does not depend on the deformation history, then the hyperelastic constitutive equation describing the nonlinear elastic and compressible properties of rubber [45,46] is

(1) $$P=\frac{\partial W(F)}{\partial F},$$

where P is the I. Piola-Kirchhoff stress tensor, and W(F) is the strain energy density function. The latter can be decomposed into two terms [16,47]

(2) $$W(F)={W_{D}\left(C\right)+W}_{V}\left(J\right),$$

where J=detF, C is the right Couchy-Green deformation tensor and $W_{V}\left(J\right)$ is the volumetric, while $W_{D}\left(C\right)$ is the deviatoric (no change in the volume) strain energy density function.

### 2.1. Hyperelastic Material Models

Polynomial formulas for the deviatoric strain energy density function can be used to describe the phenomenological models using the ${\bar{I}}_{1},{\bar{I}}_{2}$ scalar invariants of the Cauchy-Green deformation tensor, as originally proposed by Rivlin [19]. Without claiming to be exhaustive, the material models Mooney-Rivlin, Yeoh, Arruda-Boyce, and Gent are available within the polynomial formula that uses invariants. The polynomial model of the strain energy density function for incompressible material is [25]

(3) $$W_{D}\left({\bar{I}}_{1},{\bar{I}}_{2}\right)=\sum_{i+j=1}^{H}c_{ij}{\left({\bar{I}}_{1}-3\right)}^{i}{\left({\bar{I}}_{2}-3\right)}^{j},$$

where $c_{ij}$ material constants need to be characterized. Another approach, proposed by Valanis [48] and Ogden [21], describes the deviatoric strain energy density function using $\lambda_{1},\lambda_{2},\lambda_{3}$ principal stretches.

#### 2.1.1. Mooney-Rivlin Model

The Mooney-Rivlin formula has good curve fitting because of the various order models that are available when choosing how many terms to include in the polynomial formula. The values H=2 and $c_{20}=c_{02}=0$ could be inserted into Equation (3) to derive the three-term Mooney-Rivlin model [19,20]

(4) $$W_{D,MR3}\left({\bar{I}}_{1},{\bar{I}}_{2}\right)=W_{D,MR3}\left(c_{10},c_{01},c_{11}\right)=c_{10}\left({\bar{I}}_{1}-3\right)+c_{01}\left({\bar{I}}_{2}-3\right)+c_{11}\left({\bar{I}}_{1}-3\right)\left({\bar{I}}_{2}-3\right).$$

#### 2.1.2. Yeoh Model

Numerous research papers [22,49,50] demonstrate that the deviatoric strain energy for most elastomers, including rubber, depends significantly less on the ${\bar{I}}_{2}$ second scalar invariant of the Cauchy-Green deformation tensor than on the ${\bar{I}}_{1}$. Thus, the Yeoh model could be written using the third-order polynom [22]

(5) $${W_{D,Y3}\left({\bar{I}}_{1}\right)=W}_{D,Y3}\left(c_{10},c_{20},c_{30}\right)=c_{10}\left({\bar{I}}_{1}-3\right)+c_{20}{\left({\bar{I}}_{1}-3\right)}^{2}+c_{30}{\left({\bar{I}}_{1}-3\right)}^{3}.$$

#### 2.1.3. Gent Model

Given that the deviatoric strain energy is a logarithmic function of ${\bar{I}}_{1}$, the Gent model is an extension of the well-known Neo-Hooke material model [24]

(6) $$W_{D,Gent}\left({\bar{I}}_{1}\right)=W_{D,\;Gent}\left(\mu ,J_{m}\right)=-\frac{\mu J_{m}}{2}ln\left(1-\frac{{\bar{I}}_{1}-3}{J_{m}}\right),$$

where μ is the shear modulus and $J_{m}$ is the dimensionless parameter that controls the finite extensibility. This extension makes the Gent model more suitable for describing the responses of elastomer-like materials to large deformations.

#### 2.1.4. Ogden Model

A more accurate approximation in large strain ranges is provided by Ogden [21] using the principal strains directly in the polynomial model

(7) $$W_{D,Ogden}\left(\lambda_{1},\lambda_{2},\lambda_{3}\right)=W_{D,Ogden}\left(\mu ,\alpha \right)=\sum_{k=1}^{H}\;\frac{2\mu_{k}}{\alpha_{k}^{2}}\left(\lambda_{1}^{\alpha_{k}}+\lambda_{2}^{\alpha_{k}}+\lambda_{3}^{\alpha_{k}}-3\right),$$

where $\lambda_{1},\lambda_{2},\lambda_{3}$ are the principal stretches, $\mu_{k}$ is the shear modulus and $\alpha_{k}$ is the dimensionless parameter. During the investigations, the commonly used H=3 formula was used.

#### 2.1.5. Arruda-Boyce Model

Arruda and Boyce used a micromechanical model and statistical mechanics methods to predict the response of the elastomer [23]

(8) $$W_{D,AB}\left({\bar{I}}_{1}\right){=W}_{D,AB}\left(\mu ,\lambda_{L}\right)=\mu \sum_{i=1}^{5}\;\frac{c_{i}}{\lambda_{L}^{2i-2}}\left({\bar{I}}_{1}^{i}\;-3^{i}\right),\left[c_{1},c_{2},c_{3},c_{4},c_{5}\right]=\left[\frac{1}{2},\;\frac{1}{20},\frac{11}{1050},\frac{19}{7050},\frac{519}{673750}\right],$$

where μ is the shear modulus and $\lambda_{L}$ is the limit of stretch.

### 2.2. Modelling the Volumetric Strain Energy Density Function

The additions in the mixture cause rubber vulcanizates to behave as nearly incompressible material under hydrostatic pressure. The formula most commonly used to express the volumetric strain energy density function to model this behaviour is [51]

(9) $$W_{V}\left(J\right)=\frac{\kappa }{2}{\left(J-1\right)}^{2},$$

where κ is the bulk modulus, which is a true material coefficient. It is recommended that during the finite element analysis of a rubber bumper subjected to compressive load, the value κ=1000 [MPa] be used, based on my prior tests [52].

### 2.3. Drucker’s Stability

Only the nonlinear stress-strain characteristic satisfying the mathematical criteria is suitable for the material according to Drucker’s stability [53,54]. Examining Drucker’s first stability criterion within the framework of this study is crucial. It stipulates that an increase in the external influence on the body (such as time, temperature, or load) must result in a positive change in the internal energy. Therefore, energy cannot be released, so the following criterion for the inner product of the tensors has to be fulfilled

(10) $$\Delta \left(JT\right)\cdot \cdot \Delta A_{t}\geq 0,$$

where T is the Cauchy stress tensor, while $A_{t}$ is the logarithmic strain tensor. Under tensile load, hyperelastic material models remain Drucker’s stable even at large strains; however, in other load cases, they may become unstable even at small strains. Therefore, the stability of the material model response must be examined for the common load cases that are also used during material tests, to a pre-selected range of strain [25]. To investigate the stability of the material models, the range of strain based on Table 1 was selected, considering the deformation range experienced during the rubber product operation.

$-0.5\leq \varepsilon_{ten}\leq 0.5$ $-0.5\leq \varepsilon_{psh}\leq 0.5$ $-0.5\leq \varepsilon_{bia}\leq 0.5$ $0<\varepsilon_{sh}\leq 0.5$ $-0.1\leq \varepsilon_{vol}\leq 0.01$

## 3. Calibration of Hyperelastic Material Model

### 3.1. Compression Test of the Rubber Bumper

The compression test of the bumper was performed according to test conditions specified in Table 2 using an Instron 8801 (Norwood, MA, USA) uniaxial test machine.

**Figure 1:**
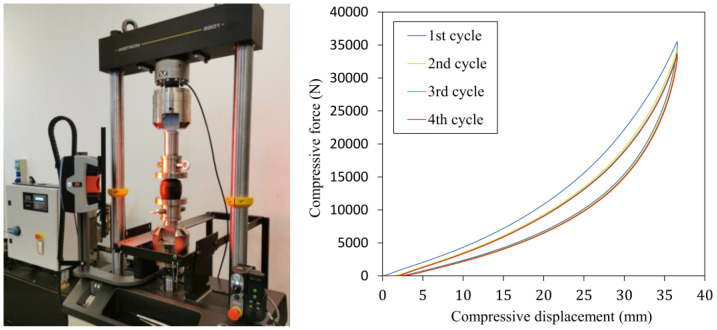
Uniaxial compression test setup and the measured characteristics of the rubber bumper.

The measured compressive force-displacement characteristics in Figure 1 show the differences between the first and second cycles due to the viscous and Mullins effect, which no longer cause significant changes in the additional loading cycles. The rubber bumper working characteristic was determined using the data points of the fourth half cycle.

### 3.2. Compressive Stress-Strain Characteristics of the Rubber

**Figure 2:**
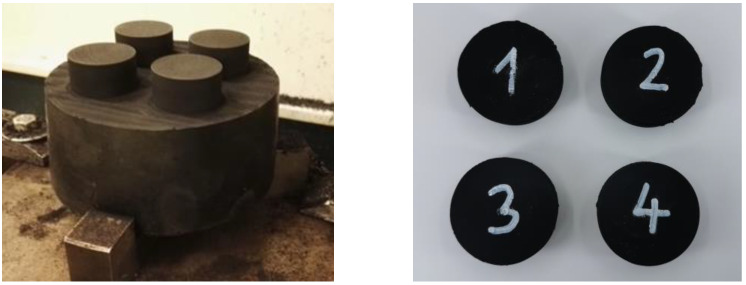
The sampled specimens and their location within the rubber product.

The rubber bumper material is a blend of natural rubber (NR) and styrene-butadiene rubber (SBR); however, the blend ratio and the additional rubber compound elements are a trade secret. Therefore, rubber samples were machined out of the product to test the material behaviour according to ISO 23529 using a milling machine. Figure 2 illustrates the location of sampling within the product and the prepared specimens, which have a diameter of 29 ± 0.5 (mm) and a height of 12 ± 0.5 (mm). As a reference to the rubber compound, the Shore hardness was measured according to the ISO 48–4 standard using a BAREISS Digi-Test II (Stouffville, ON, Canada) durometer, which is 77 Shore A for the test specimens and 78 Shore A for the product.

The compression test of the rubber specimens was performed according to the ISO 7743 ‘A’ method and the test conditions specified in Table 3. The tests were performed using an Instron 68TM-10 (Norwood, MA, USA) uniaxial test machine and the Instron AVE 2 non-contacting video extensometer. The operating rate of the traverse was calculated based on the strain rate used for the compression test of the rubber bumper.

**Figure 3:**
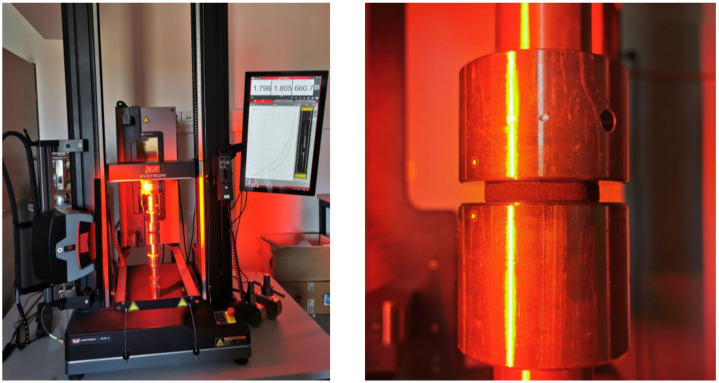
Uniaxial compression test setup and the deformation state of the specimen under maximum load.

The measurement setup is shown in Figure 3 where to reduce friction, the compression platens were prepared by mechanical polishing and PTFE (polytetrafluoroethylene) oil lubricant. Figure 3 shows the deformation state of the specimen under maximum load, based on which it can be inferred that the barrelling effect as a result of friction during the compression test is minor.

In this instance, there is an almost linear relationship between the displacement and force up to approximately 15% compressive deformation due to the specimen geometry and the test conditions can both be considered ideal. This behaviour was taken into account to correct data points that differ from linear caused by geometric errors. Assuming frictionless sliding between the rubber specimen and compression platens, the stress is homogeneous, hence the engineering stress

(11) $$\sigma_{e}=\frac{F}{A_{o}},$$

where F is the compressive force, $A_{o}$ is the original cross-sectional area. The engineering strain is expressed as the ratio of the change in dimension ΔL to the original dimension $L_{o}$ in the direction of the applied compressive load

(12) $$\varepsilon_{e}=\frac{\Delta L}{L_{o}}=\frac{L-L_{o}}{L_{o}}=\frac{L}{L_{o}}-1=\lambda -1.$$

The true strain, which is the change in instantaneous dimension L in the direction of the applied compressive load over time

(13) $$\varepsilon_{t}=\int_{L_{0}}^{L}\frac{dL}{L}={\left[\ln L\right]}_{L_{0}}^{L}=\ln L-\ln L_{0}=\ln\frac{L}{L_{0}}=\ln\left(1+\varepsilon_{e}\right)=\ln\left(\lambda \right).$$

The rubber behaves as a nearly incompressible material; thus, $\lambda_{1}\lambda_{2}\lambda_{3}\approx 1$. It allows the expression of the instantaneous cross-section A, and thus the true stress can be written

(14) $$\sigma_{t}=\frac{F}{A}=\frac{A_{0}F}{A_{0}A}=\sigma_{e}\frac{A_{0}}{A}=\sigma_{e}\frac{A_{0}\lambda }{A_{0}}=\sigma_{e}\lambda =\sigma_{e}\left(1+\varepsilon_{e}\right).$$

**Figure 4:**
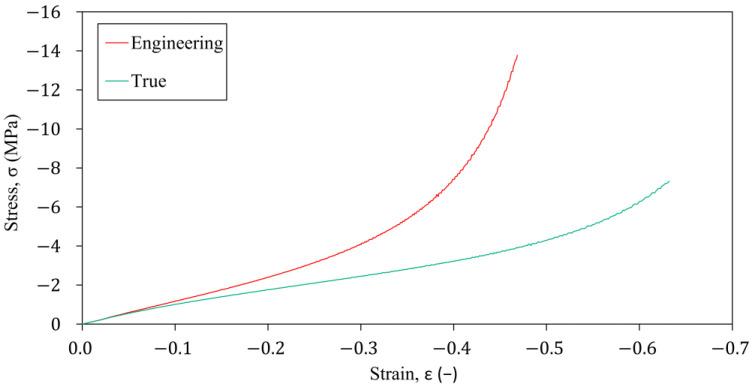
The engineering and true σ-ε characteristics under uniaxial compression test for specimen number 1.

Using Equations (11)–(14), Figure 4 shows the calculated characteristics for specimen number 1, where an inflexion point can be found in the true stress-strain curve. Therefore, a higher-order polynomial form was selected for the hyperelastic material model wherever it was available.

**Figure 5:**
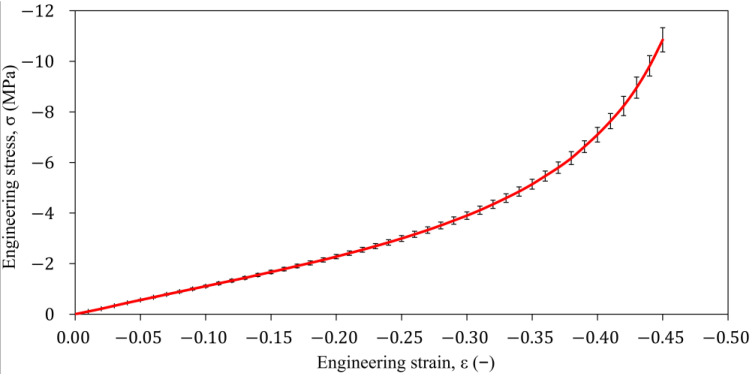
The average engineering σ-ε characteristic under uniaxial compression test for the four rubber specimens.

The stresses were calculated for the strain ε={0:0.01:0.45} by linear interpolation between the measured stress values. This was undertaken to be able to calculate the average engineering stress-strain characteristic of the four rubber specimens and to reduce the points in the data set. The result is shown in Figure 5 and Table A1.

### 3.3. Fitting the Material Parameters of the Phenomenological Hyperelastic Models Describing the Deviatoric Strain Energy Density Function

**Figure 6:**
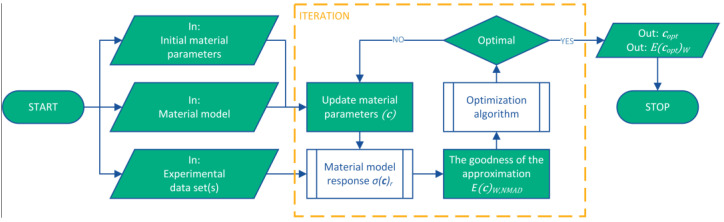
The process of curve-fitting method to find the hyperelastic material parameters.

The selection of the material parameters of the hyperelastic model is an optimization task, where the objective function is given as the difference between the measured stress-strain characteristic and the one predicted by the material model. Let C be the set of the c vector of variable material coefficients and $\sigma_{i,e}$ the measured stress for the i-th deformation state, while the ${\sigma \left(c\right)}_{i,r}$ the stress predicted by the hyperelastic model. Minimizing the objective function, which is calculated as a normalized mean absolute difference (NMAD), is one way to find the $c_{opt}$, the vector of optimal material parameters

(15) $${E\left(c_{opt}\right)}_{W,NMAD}=\underset{c\in C}{\min}\frac{1}{N}\sum_{i=1}^{N}\left|\frac{{\sigma \left(c\right)}_{i,r}-\sigma_{i,e}}{\sigma_{i,e}}\right|100\%,$$

where every pair of the stress-strain data set is taken into account with the same weight under the curve fitting process. The process shown in Figure 6 is called the curve-fitting method and is used to find hyperelastic material parameters. The average engineering stress-strain data set from the uniaxial compression test, shown in Figure 5 and Table A1, was used as input for the curve-fitting process.

Table 4 lists the material parameters that were determined by fitting the phenomenological material models Mooney-Rivlin, Yeoh, Gent, Arruda-Boyce, and Ogden. Drucker’s stability test of the material models was performed for the load modes given in Table 1; only the Ogden shows unstable behaviour when the biaxial compressive load mode exceeds the $\varepsilon_{bia}=-0.42$ strain value.

### 3.4. Verification of Material Models Using the Finite Element Model of the Compression Test

The finite element simulation of the uniaxial compression test with the material parameters in Table 4 was used to evaluate the accuracy of the fitted material models. Axisymmetric linear quadrilateral element (PLANE183) with the settings shown in Table 5 was chosen for the finite element discretization due to the isotropic material, the axisymmetric geometry and boundary conditions. It should be noted that during the compression tests, it is assumed that the stress distribution in the rubber specimen is homogeneous. Consequently, the stress response can be obtained precisely with just one element.

**Figure 7:**
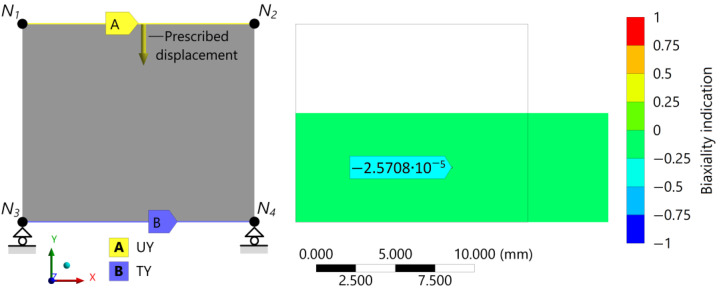
The 2D axisymmetric discretized model of the compression specimen indicating the boundary conditions (prescribed displacement *UY* in direction −*y*, roller support *TY*) and the post-processed deformation state under 45% prescribed compressive load with the distribution of the stress biaxiality in the contour plot.

At nodes 1 and 2 on the upper edge of the test specimen, the prescribed displacement of 5.625 (mm) (45% compression) in direction −*y* is applied as a load; see *UY* in Figure 7. Furthermore, to model the frictionless connection between the specimen and the compression platen, the displacement is constrained in the *y* direction at nodes 3 and 4 on the lower edge; see *TY* in Figure 7.

Post-processing the biaxiality indication could be one way to interpret the strain state of a rubber product under compressive load. The ratio of the smaller to larger principal stress—ignoring the principal stress closest to zero—defines the biaxiality indication. Hence, the locations of the uniaxial stress state are reported by a value of zero, pure shear by minus one, and biaxial by one. According to Figure 7, the numerical test results of the compression test show nearly zero biaxiality indication to the whole specimen, which was expected under the uniaxial load case.

**Figure 8:**
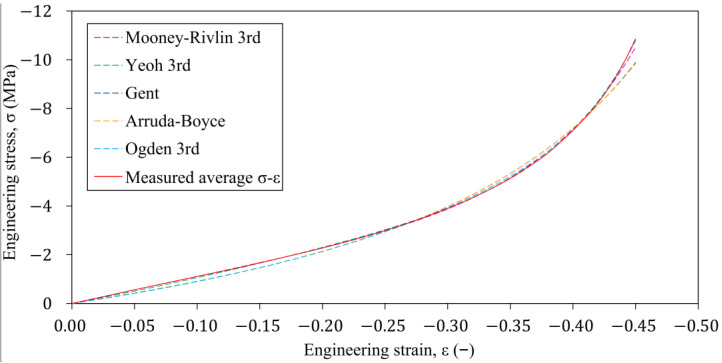
Comparison of the different hyperelastic models’ prediction with the laboratory test results of the average engineering σ-ε characteristic.

The ANSYS Mechanical solver and Newton-Raphson numerical technique were used to solve the nonlinear problem. The number of sub-steps was selected in the analysis setup to match the number of data points on the average engineering σ-ε characteristic. The engineering stress-strain characteristics were computed numerically using the fitted hyperelastic models; see Figure 8 for the results.

**Figure 9:**
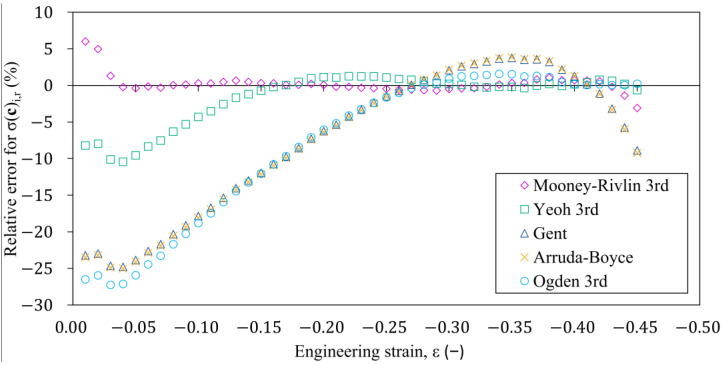
The variation in the relative error of the different hyperelastic models compared to the average σ-ε characteristic measured on the specimens.

Figure 9 shows the errors of the stresses predicted to the specific elongation values ε={0:0.01:0.45} of the fitted hyperelastic models that were determined relative to the stresses measured on the laboratory test. Based on these results, in the range of strains above 10%, the Yeoh and Mooney-Rivlin material models approximate the measured stress-strain characteristic of the rubber within the error of less than 5%. The prediction of the Mooney-Rivlin and Yeoh material models differ significantly from rubber’s linear behaviour in the strain region below 10%. This difference can be attributed to the polynomial function approximation used to approximate the deformation energy density. The reason for this phenomenon can be caused by the use of the polynomial form to approximate the deviatoric strain energy density function.

**Figure 10:**
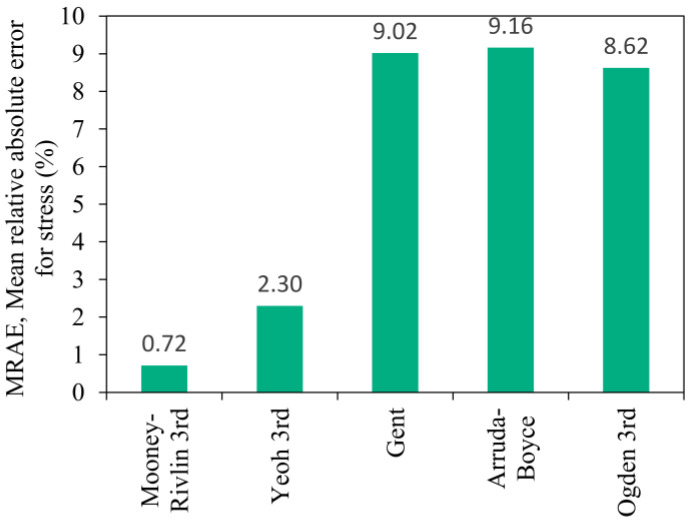
The mean relative absolute error of the hyperelastic model prediction capability relative to the average σ-ε characteristic.

Figure 10 shows the mean relative absolute error (MRAE) calculated using the values of the relative errors previously determined in Figure 9 can be used to determine generally the goodness of the material model’s prediction capability. The Gent, Arruda-Boyce and Ogden material models have close to 10% MRAE, mostly because the predicted stress values in the upper strain range are more accurate. Thus, it is not recommended to use these models to predict the material stress-strain behaviour over the whole range of a given strain. The results showed that the Mooney-Rivlin and Yeoh material models can predict the material response to the compressive load with less than 2.5% MRAE.

## 4. Verification of Material Models Using the Finite Element Model of the Rubber Bumper Compression Test

**Figure 11:**
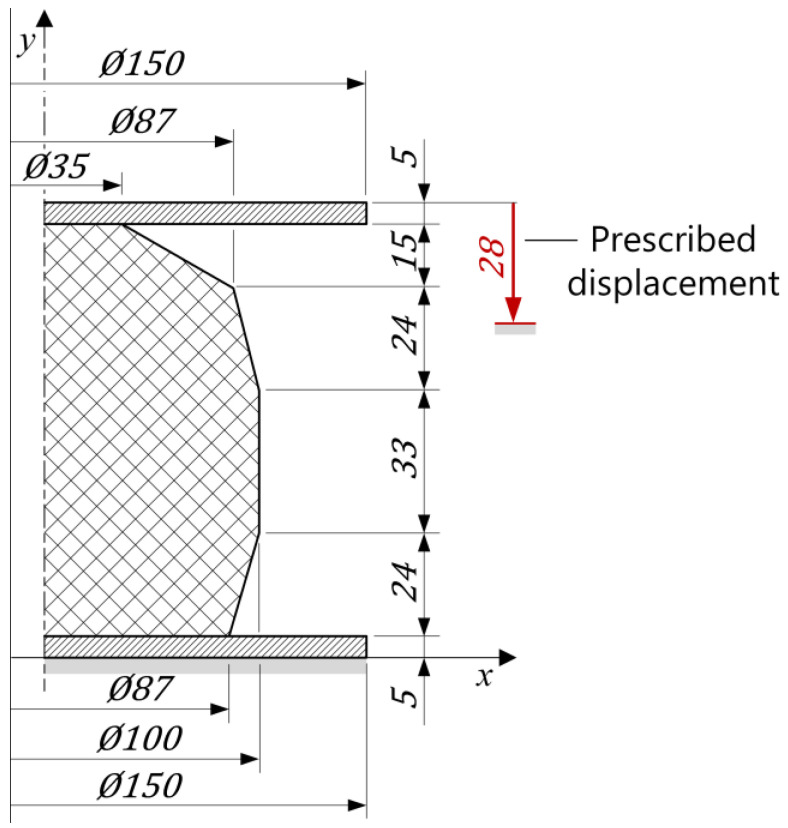
Geometric dimensions and the meridian section of the compression platens and the rubber product.

The finite element analysis of the compression test of the rubber bumper was used to evaluate the prediction capability of the hyperelastic models, fitted for the compression test, for different strain states. The inhomogeneous strain state within the rubber product under compressive load is due to the complex geometry, large deformation and connections. This investigation requires post-processing of the numerical analysis results for each of the material models to determine the predicted working characteristics of the product. These results must then be compared to the laboratory test results shown in Figure 1. Figure 11 shows the geometric dimensions and the meridian section of the compression platens and the product.

**Figure 12:**
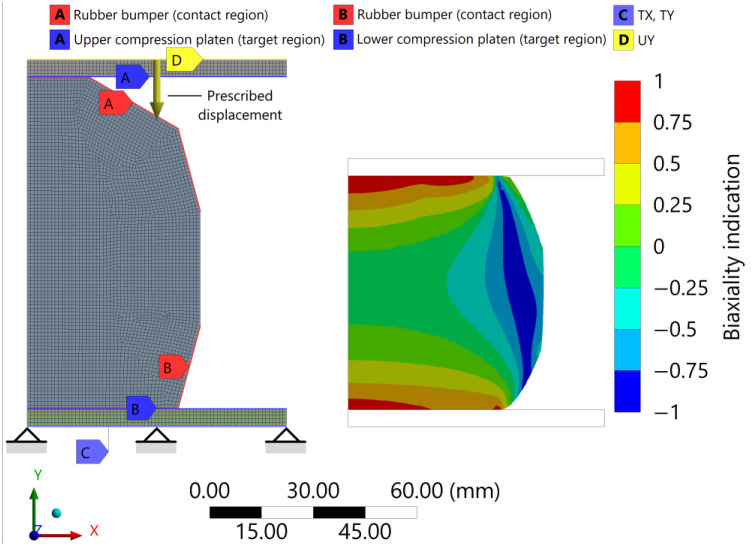
The 2D axisymmetric discretized model of the rubber bumper indicating the boundary conditions and contact regions and the post-processed deformation state under 30% prescribed compressive load with the distribution of the stress biaxiality in the contour plot.

**Figure A1:**
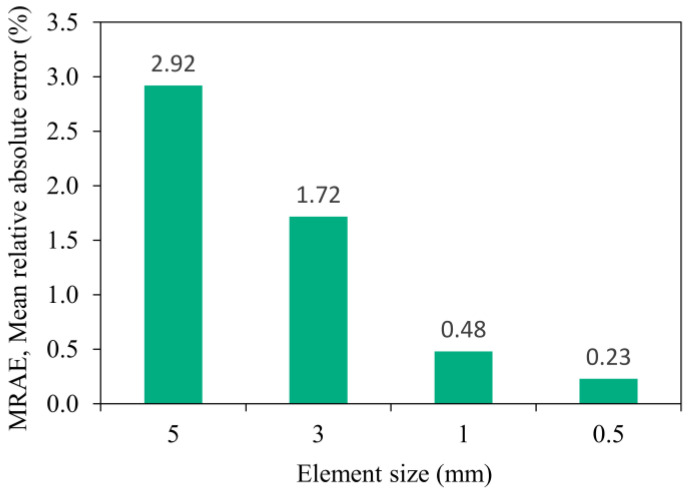
The sensitivity of the compressive force to changes in mesh density.

Mesh locking under large deformations is a phenomenon that can be observed in meshes discretized with nearly incompressible material. In such a case, for the meshing, linear quadrilateral elements that are less prone to mesh locking must be used. To reduce the numerical error in the determination of the operating characteristic of the rubber bumper a force convergence test was carried out while changing the mesh density. Investigating Figure A1, by choosing an element size of 1 (mm), the numerical solution error is under 0.5%. Table 6 contains the settings for the finite element discretization, while the resulting finite element mesh for the bumper is seen in Figure 12.

The rubber bumper contacts the upper and lower compression platens as it operates while resulting in a 30% reduction in height. Hence, *A* and *B* frictional edge-to-edge connections were defined, where the contact elements were created on the rubber bumper, while the target elements were created on the compression platens according to Figure 12. The static friction coefficient was selected as ${µ}_{s}=0.6$ [47]; thus, the state of contact between the contacting elements is mostly sticking under the compression test. At the edge of the upper compression platen, the prescribed displacement of 28 (mm) in direction −*y* is applied as a load; see *UY* in Figure 12. Furthermore, the displacement is constrained in the *x* and *y* directions at the edge of the lower compression platen; see *TX*, *TY*.

**Figure 13:**
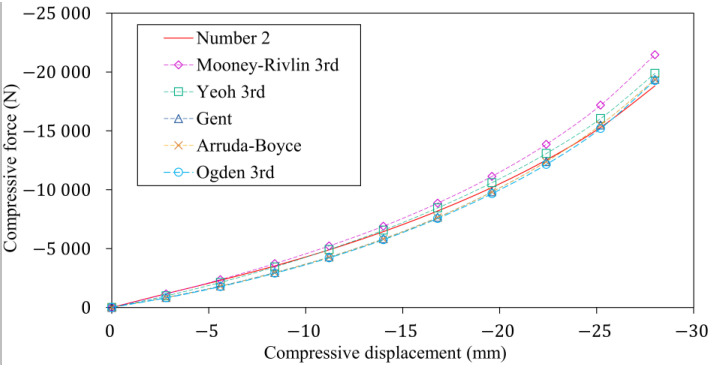
The laboratory and the numerical test results of the rubber bumper working characteristic.

After the converged nonlinear numerical analysis, Figure 12 shows the deformation state under maximum compressive displacement, allowing the appropriate operation of the discretized model and connections to be verified. Additionally, the stress biaxiality indication in the contour plot shows, in line with previous intuition, that the stress state of the rubber bumper varies significantly by location. In a significant part of the inside of the rubber bumper, the state of stress is nearly uniaxial; however, close to the compression platen where the contact is sticking, it is biaxial, while shear also occurs near the diameter. In locations where the biaxiality indicator deviates significantly from zero, the prediction of the material models may differ significantly from the material real behaviour, as the fitted hyperelastic material models lack information for load cases other than the uniaxial compression. After running the finite element analysis with the different hyperelastic models, Figure 13 shows to what extent the predicted characteristics differ from the measured one.

**Figure 14:**
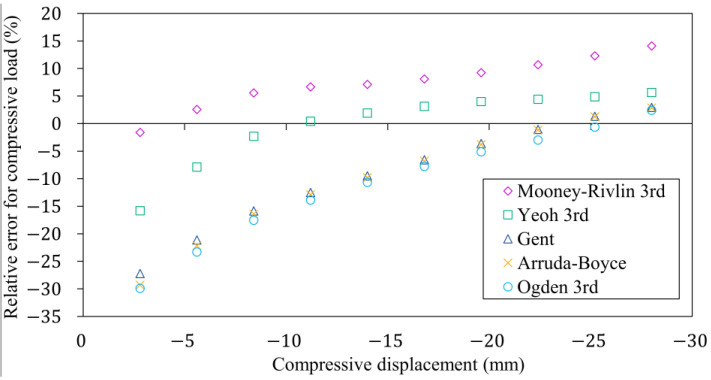
The variation in the relative error of the different hyperelastic models compared to the characteristic measured on the rubber bumper.

Figure 14 shows the error of the reaction forces predicted by the hyperelastic models for ten uniformly distributed compression levels relative to compressive force from the laboratory test. The Gent, Arruda-Boyce and Ogden material models have large deviations in the simulated compressive force under almost the whole compressive displacement. In comparison to the characteristics measured and simulated by the Yeoh material model, the numerical result analysed with the Mooney-Rivlin material model shows a stiffer behaviour in almost the whole compressive displacement. This is due to the strain energy based hyperelastic model fitted to the uniaxial compressive load case, which cannot accurately predict material responses different from the known strain state.

**Figure 15:**
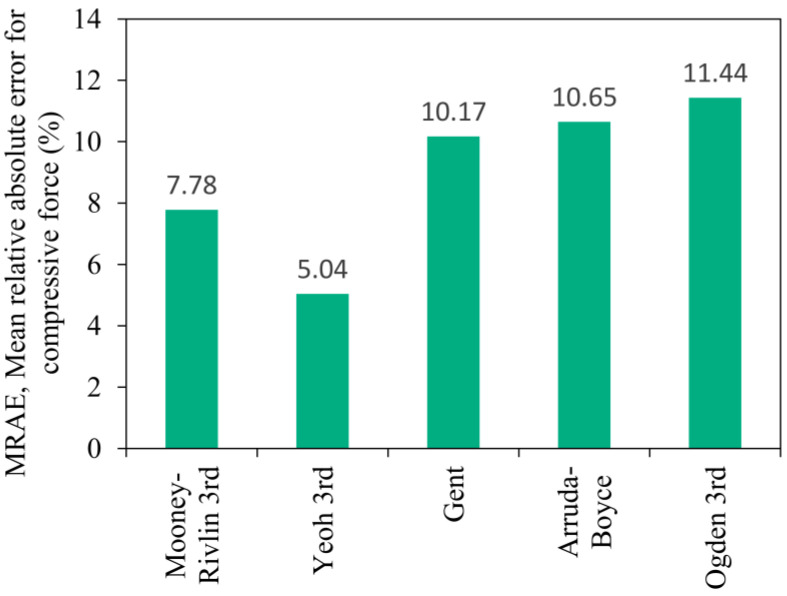
The mean relative absolute error of the hyperelastic models compared to the characteristic of the rubber product.

A deviation of more than 10% can be observed when investigating the MRAE shown in Figure 15 for the Gent, Arruda-Boyce, and Ogden models. In comparison to the values observed during the finite element run of the compression test, the MRAE of the Yeoh material model increased by 3% and the Mooney-Rivlin increased by 7% based on simulation results shown in Figure 10. Nevertheless, the material response to an inhomogeneous strain state can be estimated by the Mooney-Rivlin and Yeoh material models with an error significantly below 10%, which is acceptable in engineering simulation problems.

## 5. Conclusions

Designing rubber products requires special consideration of the numerical discretization process due to the nonlinear behaviours (material nonlinearity, large deformation, connections). Modelling considerations were presented for the finite element analysis of the rubber bumper. If the rubber behaviour is only determined for the specific load of the product, which, in the case of rubber bumpers, is the compression, the time needed for the laboratory test can be significantly decreased.

Based on the foregoing, my research aimed at fitting and selecting a hyperelastic material model suitable for the finite element modelling of the working characteristic of a rubber product under compressive load. The Mullins effect as well as the viscoelastic and hysteretic material behaviours were not modelled because the time dependence was not taken into consideration during the product testing of the rubber bumpers.

Rubber samples for compression tests were machined out of the product because the content of the rubber compounds is a trade secret. The stress-strain characteristics of the uniaxial compression test of rubber samples were used to fit the hyperelastic material models (Mooney-Rivlin, Yeoh, Gent, Arruda-Boyce, and Ogden). The results showed that the Mooney-Rivlin and Yeoh material models fitted for the uniaxial compression data set can predict the material response up to 45% uniaxial compressive deformation with less than 2.5% MRAE if the stress state is homogeneous. Nevertheless, the Gent, Arruda-Boyce and Ogden material models are unable to properly predict material response even in a homogeneous stress state. The resulting 10% MRAE is mostly because the predicted stress values in the upper strain range are more accurate; therefore, the use of these material models in the investigated strain range is not recommended in the case of compressive load. The laboratory and numerical tests of the rubber bumper were used to determine the accuracy of the material models if the stress state is changing. The distribution of the stress biaxiality in the contour plot is a good measure to determine which load mode is required for the curve fitting of the hyperelastic model. The results showed that if only uniaxial compression test data are available for the curve fitting of the material model, the Yeoh model performs the best in predicting how the rubber product behaves under compressive load and complex strain state.

The research pointed out that the constitutive modelling error in complex strain state could be significantly below 10% when using only the uniaxial compression data set to calibrate the Yeoh or Mooney Rivlin material models. This is acceptable in nonlinear engineering simulation problems. Non-standard mechanical tests like simple shear and biaxial could therefore be eliminated, saving a significant amount of time and money, which would help increase market competitiveness.

The developed process opens up numerous new research possibilities. One area is the investigation of other load modes or different strain ranges, for which the product can be selected using the introduced biaxiality measure. Other hyperelastic materials like 3D-printable thermoplastic polyurethane blends could be investigated to shorten the specimen preparation and the product manufacturing time. Using the introduced biaxiality measure, an extension of the research could be to develop a strain distribution-based material model calibration method.

## Figures and Tables

**Table 1:** The selected range of strains for different modes of load to examine Drucker’s stability.

Mode of Load	Range of Strain
Uniaxial compression-tension	$-0.5\leq \varepsilon_{ten}\leq 0.5$
Pure shear	$-0.5\leq \varepsilon_{psh}\leq 0.5$
Biaxial compression-tension	$-0.5\leq \varepsilon_{bia}\leq 0.5$
Simple shear	$0<\varepsilon_{sh}\leq 0.5$
Volumetric compression-tension	$-0.1\leq \varepsilon_{vol}\leq 0.01$

**Table 2:** Test conditions of the uniaxial compression of the rubber bumper.

Temperature and Relative Humidity of the Test	25 (°C); 50%
The number, magnitude and speed of the preload cycles	3 cycles; 35 mm; 50 (mm∙min^−1^)
The number, magnitude and speed of the measured cycle	4th cycle; 35 (mm); 50 (mm∙min^−1^)
Test setup	Polished compression platen without lubrication

**Table 3:** Test conditions of the uniaxial compression of the cylindrical rubber specimens.

Temperature and Relative Humidity of the Test	25 (°C); 50%
The number, magnitude and speed of the preload cycles	3 cycles; 6.4 (mm); 6.76 (mm∙min^−1^)
The number, magnitude and speed of the measured cycle	4th cycle; 6.4 (mm); 6.76 (mm∙min^−1^)
Test setup	Polished and lubricated compression platens

**Table 4:** Hyperelastic material parameters found by the curve fitting method.

Hyperelastic Model	Material Parameters	$\mathrm{Fitting}\;\mathrm{Error}\;\;{E\left(c_{opt}\right)}_{W,NMAD}$	Drucker’s Stability
Mooney-Rivlin 3rd	$c_{10}=4.33252\;\left(MPa\right)$	0.721%	stable
	$c_{01}=2.44690\;\left(MPa\right)$		
	$c_{11}=0.65050\;\left(MPa\right)$		
Yeoh 3rd	$c_{10}=1.61168\left(MPa\right)$	0.752%	stable
	$c_{20}=0.46206\left(MPa\right)$		
	$c_{30}=0.45899\left(MPa\right)$		
Gent	$\mu =2.69532\;\left(MPa\right)$	4.711%	stable
	$J_{m}=3.77895\;\left(-\right)$		
Arruda-Boyce	$\mu =1.51128\left(MPa\right)$	4.833%	stable
	$\lambda_{L}=1.28813\left(-\right)$		
Ogden 3rd	$\mu_{1}=-1.07018\;\left(MPa\right)$	2.830%	not stable for biaxial compression $\varepsilon_{bia}<-0.42$
	$\mu_{2}=0.00018\;\left(MPa\right)$		
	$\mu_{3}=43.17404\;\left(MPa\right)$		
	$\alpha_{1}=0.00018\;\left(-\right)$		
	$\alpha_{1}=30.15348\;\left(-\right)$		
	$\alpha_{1}=0.11882\;\left(-\right)$		

**Table 5:** Settings for the finite element discretization.

Element Type, Order and Shape	Axisymmetric Linear Quadrilateral
Number of elements	1
Material model	Isotropic, hyperelastic according to Table 4.,$\kappa =1000\;\left(MPa\right)$ [52]

**Table 6:** Settings for the finite element discretization.

Element Type, Order and Shape	Axisymmetric Linear Quadrilateral
Element size	1 (mm)
Material model for the compression platens	Isotropic, linearly elastic $E=2{\cdot 10}^{5}\left(MPa\right),\;\nu =0.3$
Material model for the bumper	Isotropic, hyperelastic according to Table 4$.,\;\kappa =1000\;\left(MPa\right)$ [52]

## Data Availability

The data presented in this study are available on request from the corresponding author.

## Appendix A

**Table A1:** Stress values are interpolated to discrete strain values between the points of the engineering σ-ε characteristic curve of the specimens measured by the uniaxial compression test.

$\varepsilon_{e}(-)$	$\sigma_{e}\left(MPa\right)$						
	No.1	No.2	No.3	No.4	Average	Median	Standard Deviation
−0.01	−0.11	−0.10	−0.11	−0.11	−0.106	−0.107	0.004
−0.02	−0.22	−0.21	−0.22	−0.21	−0.214	−0.214	0.006
−0.03	−0.35	−0.33	−0.32	−0.32	−0.332	−0.329	0.013
−0.04	−0.47	−0.45	−0.44	−0.43	−0.448	−0.443	0.018
−0.05	−0.59	−0.56	−0.55	−0.54	−0.560	−0.556	0.023
−0.06	−0.71	−0.67	−0.66	−0.64	−0.670	−0.665	0.030
−0.07	−0.82	−0.78	−0.77	−0.75	−0.781	−0.775	0.030
−0.08	−0.94	−0.89	−0.87	−0.86	−0.889	−0.881	0.036
−0.09	−1.05	−1.01	−0.98	−0.96	−0.998	−0.992	0.041
−0.10	−1.17	−1.11	−1.08	−1.07	−1.107	−1.096	0.044
−0.11	−1.29	−1.22	−1.19	−1.17	−1.218	−1.205	0.051
−0.12	−1.40	−1.33	−1.30	−1.27	−1.326	−1.313	0.056
−0.13	−1.51	−1.44	−1.41	−1.39	−1.435	−1.423	0.052
−0.14	−1.63	−1.56	−1.52	−1.50	−1.550	−1.539	0.059
−0.15	−1.76	−1.67	−1.64	−1.60	−1.667	−1.653	0.067
−0.16	−1.87	−1.80	−1.75	−1.72	−1.784	−1.773	0.069
−0.17	−2.00	−1.91	−1.88	−1.83	−1.906	−1.893	0.071
−0.18	−2.14	−2.03	−1.99	−1.95	−2.026	−2.008	0.081
−0.19	−2.26	−2.15	−2.12	−2.06	−2.147	−2.134	0.083
−0.20	−2.39	−2.28	−2.23	−2.20	−2.276	−2.258	0.085
−0.21	−2.53	−2.42	−2.37	−2.33	−2.412	−2.396	0.088
−0.22	−2.68	−2.55	−2.50	−2.45	−2.547	−2.528	0.098
−0.23	−2.82	−2.70	−2.65	−2.59	−2.691	−2.675	0.099
−0.24	−2.98	−2.85	−2.79	−2.73	−2.838	−2.822	0.108
−0.25	−3.15	−3.00	−2.95	−2.88	−2.994	−2.976	0.115
−0.26	−3.32	−3.18	−3.10	−3.03	−3.159	−3.140	0.122
−0.27	−3.50	−3.33	−3.29	−3.19	−3.327	−3.309	0.128
−0.28	−3.68	−3.53	−3.46	−3.36	−3.509	−3.495	0.135
−0.29	−3.89	−3.72	−3.66	−3.54	−3.703	−3.689	0.145
−0.30	−4.09	−3.91	−3.86	−3.74	−3.899	−3.882	0.147
−0.31	−4.33	−4.13	−4.06	−3.94	−4.113	−4.091	0.163
−0.32	−4.56	−4.37	−4.29	−4.17	−4.347	−4.329	0.162
−0.33	−4.81	−4.62	−4.53	−4.41	−4.593	−4.577	0.170
−0.34	−5.09	−4.88	−4.78	−4.65	−4.852	−4.834	0.184
−0.35	−5.38	−5.17	−5.09	−4.92	−5.140	−5.130	0.194
−0.36	−5.72	−5.50	−5.41	−5.23	−5.464	−5.453	0.200
−0.37	−6.09	−5.82	−5.72	−5.54	−5.795	−5.773	0.227
−0.38	−6.48	−6.23	−6.11	−5.87	−6.171	−6.169	0.255
−0.39	−6.89	−6.68	−6.59	−6.33	−6.624	−6.635	0.234
−0.40	−7.43	−7.18	−7.07	−6.73	−7.101	−7.126	0.291
−0.41	−7.95	−7.75	−7.61	−7.24	−7.635	−7.679	0.301
−0.42	−8.66	−8.37	−8.15	−7.76	−8.234	−8.258	0.382
−0.43	−9.39	−9.17	−8.85	−8.43	−8.959	−9.007	0.419
−0.44	−10.17	−10.08	−9.76	−9.27	−9.820	−9.920	0.402
−0.45	−11.19	−11.21	−10.82	−10.18	−10.851	−11.006	0.480

## Appendix B

Every tenth sub-step of the numerical analysis was used to perform the force convergence test, which involved splitting the prescribed displacement of 28 mm into ten equal parts. Figure A1 shows the investigated element sizes. The Mooney-Rivlin hyperelastic material model was used for modelling in all instances, and the compressive force values obtained with an element size of 0.2 (mm) were used as the relative values. Based on the results, Figure A1 shows the mean relative absolute error of the numerical solution for different mesh densities.

## References

[1] Palička P., Huňady R., Hagara M., Lengvarský P. (2022). Optimization of Apex Shape for Mounting to the Bead Bundle Using FEM. Materials.

[2] Kudelin D.V., Nesiolovskaia T.N. (2022). Application of the Finite Element Analysis in the Design of Rubber Membranes. AIP Conf. Proc..

[3] Jin L., Li S., Cheng Y., Liu J. (2022). A Time-Dependent Yeoh Model to Predict the Corrosion Effect of Supercritical CO_2_ on the HNBR Sealing Rubber. J. Mech. Sci. Technol..

[4] Cernuda C., Llavori I., Zavoianu A.-C., Aguirre A., Zabala A., Plaza J. Critical Analysis of the Suitability of Surrogate Models for Finite Element Method Application in Catalog-Based Suspension Bushing Design. Proceedings of the 2020 25th IEEE International Conference on Emerging Technologies and Factory Automation (ETFA).

[5] Premarathna W.A.A.S., Jayasinghe J.A.S.C., Wijesundara K.K., Gamage P., Ranatunga R.R.M.S.K., Senanayake C.D. (2021). Investigation of Design and Performance Improvements on Solid Resilient Tires through Numerical Simulation. Eng. Fail. Anal..

[6] Zheng C., Zheng X., Qin J., Liu P., Aibaibu A., Liu Y. (2021). Nonlinear Finite Element Analysis on the Sealing Performance of Rubber Packer for Hydraulic Fracturing. J. Nat. Gas Sci. Eng..

[7] Dong L., Tang Y., Tang G., Li H., Wu K., Luo W. Sealing Performance Analysis of Rubber Core of Annular BOP: FEM Simulation and Optimization to Prevent the SBZ. Petroleum.

[8] Szántó A., Hajdu S., Sziki G.Á. (2023). Optimizing Parameters for an Electrical Car Employing Vehicle Dynamics Simulation Program. Appl. Sci..

[9] Wu J., He Y., Wu K., Dai M., Xia C. (2021). The Performance Optimization of the Stripper Rubber for the Rotating Blowout Preventer Based on Experiments and Simulation. J. Pet. Sci. Eng..

[10] Hejazi F., Farahpour H., Ayyash N., Chong T. (2022). Development of a Volumetric Compression Restrainer for Structures Subjected to Vibration. J. Build. Eng..

[11] Mankovits T., Szabo T., Kocsis I., Paczelt I. (2014). Optimization of the Shape of Axi-Symmetric Rubber Bumpers. Stroj. Vestnik/Journal Mech. Eng..

[12] Mankovits T., Szabó T. (2012). Finite Element Analysis of Rubber Bumper Used in Air-Springs. Procedia Eng..

[13] Mankovits T., Kocsis I., Portik T., Szabó T., Páczelt I. (2013). Shape Design of Rubber Part Using FEM. Int. Rev. Appl. Sci. Eng..

[14] Kaya N. (2014). Shape Optimization of Rubber Bushing Using Differential Evolution Algorithm. Sci. World J..

[15] Somanath S., Marimuthu R., Krishnapillai S., Narayanan S. Transient Vibration Response Study of Moulded and Pre-Stressed Silicone Elastomer Vibration Isolators. Int. J. Dyn. Control.

[16] Bonet J., Wood R.D. (2008). Nonlinear Continuum Mechanics for Finite Element Analysis.

[17] Kozák I. (1995). Kontinuummechanika.

[18] Ward I.M., Sweeney J. (2012). Mechanical Properties of Solid Polymers.

[19] Rivlin R.S. (1948). Large Elastic Deformations of Isotropic Materials. I. Fundamental Concepts. Philos. Trans. R. Soc. London. Ser. A Math. Phys. Sci..

[20] Mooney M. (1940). A Theory of Large Elastic Deformation. J. Appl. Phys..

[21] Ogden R.W., A P.R.S.L. (1972). Large Deformation Isotropic Elasticity – on the Correlation of Theory and Experiment for Incompressible Rubberlike Solids. Proc. R. Soc. Lond. A. Math. Phys. Sci..

[22] Yeoh O.H. (1993). Some Forms of the Strain Energy Function for Rubber. Rubber Chem. Technol..

[23] Arruda E.M., Boyce M.C. (1993). A Three-Dimensional Constitutive Model for the Large Stretch Behavior of Rubber Elastic Materials. J. Mech. Phys. Solids.

[24] Gent A.N. (1996). A New Constitutive Relation for Rubber. Rubber Chem. Technol..

[25] Bergström J. (2015). Mechanics of Solid Polymers.

[26] El Yaagoubi M., Fulari G.S., Aloui S., Shetty R.R. (2021). Influence of Permanent Deformation on the Fitting Quality and the Simulation Prediction of Filled Elastomers. Int. J. Non. Linear. Mech..

[27] Aloui S., El Yaagoubi M. (2021). Determining the Compression-Equivalent Deformation of SBR-Based Rubber Material Measured in Tensile Mode Using the Finite Element Method. Appl. Mech..

[28] Treloar L.R.G. (1944). Stress-Strain Data for Vulcanized Rubber under Various Types of Deformation. Rubber Chem. Technol..

[29] Steinmann P., Hossain M., Possart G. (2012). Hyperelastic Models for Rubber-like Materials: Consistent Tangent Operators and Suitability for Treloar’s Data. Arch. Appl. Mech..

[30] Hossain M., Steinmann P. (2013). More Hyperelastic Models for Rubber-like Materials: Consistent Tangent Operators and Comparative Study. J. Mech. Behav. Mater..

[31] Marckmann G., Verron E. (2006). Comparison of Hyperelastic Models for Rubber-Like Materials. Rubber Chem. Technol..

[32] Dal H., Açıkgöz K., Badienia Y. (2021). On the Performance of Isotropic Hyperelastic Constitutive Models for Rubber-Like Materials: A State of the Art Review. Appl. Mech. Rev..

[33] Melly S.K., Liu L., Liu Y., Leng J. (2021). A Review on Material Models for Isotropic Hyperelasticity. Int. J. Mech. Syst. Dyn..

[34] He H., Zhang Q., Zhang Y., Chen J., Zhang L., Li F. (2022). A Comparative Study of 85 Hyperelastic Constitutive Models for Both Unfilled Rubber and Highly Filled Rubber Nanocomposite Material. Nano Mater. Sci..

[35] Fazekas B., Goda T.J. (2020). New Numerical Stress Solutions to Calibrate Hyper-Visco-Pseudo-Elastic Material Models Effectively. Mater. Des..

[36] Fazekas B., Goda T.J. (2021). Constitutive Modelling of Rubbers: Mullins Effect, Residual Strain, Time-Temperature Dependence. Int. J. Mech. Sci..

[37] Fazekas B., Goda T.J. (2019). Closed-Form and Numerical Stress Solution-Based Parameter Identification for Incompressible Hyper-Viscoelastic Solids Subjected to Various Loading Modes. Int. J. Mech. Sci..

[38] Fazekas B., Burkhart C., Staub S., Thielen S., Andrä H., Goda T.J., Sauer B., Koch O. (2023). Radial Shaft Seals: How Ageing in Oil and Hyper-Viscoelasticity Affect the Radial Force and the Numerically Predicted Wear. Tribol. Int..

[39] Zhao Z., Mu X., Du F. (2019). Modeling and Verification of a New Hyperelastic Model for Rubber-Like Materials. Math. Probl. Eng..

[40] Guo L., Zeng Y., Huang J., Wang Z., Li J., Han X., Xia C., Qian L. (2022). Fatigue Optimization of Rotary Control Head Rubber Core Based on Steady Sealing. Eng. Fail. Anal..

[41] Nguyen H.-D., Huang S.-C. (2021). The Uniaxial Stress–Strain Relationship of Hyperelastic Material Models of Rubber Cracks in the Platens of Papermaking Machines Based on Nonlinear Strain and Stress Measurements with the Finite Element Method. Materials.

[42] Íñiguez-Macedo S., Lostado-Lorza R., Escribano-García R., Martínez-Calvo M. (2019). Finite Element Model Updating Combined with Multi-Response Optimization for Hyper-Elastic Materials Characterization. Materials.

[43] Lei G., Chen Q., Liu Y., Jiang J. (2013). An Inverse Method to Reconstruct Complete Stiffness Information of Rubber Bushing. Adv. Mater. Sci. Eng..

[44] Yenigun B., Gkouti E., Barbaraci G., Czekanski A. (2022). Identification of Hyperelastic Material Parameters of Elastomers by Reverse Engineering Approach. Materials.

[45] Fazekas B., Goda T.J. (2020). Numerical Stress Solutions for the Accurate Calibration of Hyper-Viscoelastic Material Models of Polymer Foams. Int. J. Solids Struct..

[46] Fazekas B., Goda T.J. (2018). Determination of the Hyper-Viscoelastic Model Parameters of Open-Cell Polymer Foams and Rubber-like Materials with High Accuracy. Mater. Des..

[47] Holzapfel G.A. (2000). Nonlinear Solid Mechanics, A Continuum Approach for Engineering.

[48] Valanis K.C., Landel R.F. (1967). The Strain-Energy Function of a Hyperelastic Material in Terms of the Extension Ratios. J. Appl. Phys..

[49] Kaliske M., Rothert H. (1997). On the Finite Element Implementation of Rubber-like Materials at Finite Strains. Eng. Comput..

[50] Kawabata S., Yamashita Y., Ooyama H., Yoshida S. (1995). Mechanism of Carbon-Black Reinforcement of Rubber Vulcanizate. Rubber Chem. Technol..

[51] Horgan C.O., Murphy J.G. (2009). On the Volumetric Part of Strain-Energy Functions Used in the Constitutive Modeling of Slightly Compressible Solid Rubbers. Int. J. Solids Struct..

[52] Huri D. (2016). Incompressibility and Mesh Sensitivity Analysis in Finite Element Simulation of Rubbers. Int. Rev. Appl. Sci. Eng..

[53] Drucker D.C. (1959). A Definition of a Stable Inelastic Material. J. Appl. Mech..

[54] Hill R. (1958). A General Theory of Uniqueness and Stability in Elastic-Plastic Solids. J. Mech. Phys. Solids.

